# Antimicrobial resistance and metallo-beta-lactamase producing among commensal *Escherichia coli* isolates from healthy children of Khuzestan and Fars provinces; Iran

**DOI:** 10.1186/s12866-020-02051-8

**Published:** 2020-12-01

**Authors:** Fahimeh Mahmoodi, Seyedeh Elham Rezatofighi, Mohammad Reza Akhoond

**Affiliations:** 1 Department of Biology, Faculty of Science, Shahid Chamran University of Ahvaz, Ahvaz, Postal code: 6135743135 Iran; 2 Mathematical Sciences and Computer Faculty, Shahid Chamran University of Ahvaz, Ahvaz, Iran

**Keywords:** Commensal *Escherichia coli*, Antimicrobial resistance, Metallo-β-lactamase, Carbapenem, NDM, VIM, Conjugation

## Abstract

**Background:**

The emergence of metallo-β-lactamase (MBL)-producing isolates is alarming since they carry mobile genetic elements with great ability to spread; therefore, early detection of these isolates, particularly their reservoir, is crucial to prevent their inter- and intra-care setting dissemination and establish suitable antimicrobial therapies. The current study was designed to evaluate the frequency of antimicrobial resistance (AMR), MBL producers and identification of MBL resistance genes in *Escherichia coli* strains isolated from fecal samples of the healthy children under 3 years old. A total of 412 fecal *E. coli* isolates were collected from October 2017 to December 2018. The study population included healthy infants and children aged < 3 years who did not exhibit symptoms of any diseases, especially gastrointestinal diseases. *E. coli* isolates were assessed to determine the pattern of AMR. *E. coli* isolates were assessed to determine the pattern of AMR, the production of extended spectrum β-lactamase (ESBL) and MBL by phenotypic methods. Carbapenem-resistant isolates were investigated for the presence of MBL and carbapenemase genes, plasmid profiling, and the ability of conjugation.

**Results:**

In sum, AMR, multi-drug resistance (MDR) and ESBL production were observed in more than 54.9, 36.2 and 11.7% of commensal *E. coli* isolates, respectively. Out of six isolates resistant to imipenem and meropenem, four isolates were phenotypically detected as MBL producers. Two and one *E. coli* strains carried the *bla*_NDM-1_ and *bla*_VIM-2_ genes, respectively and were able to transmit imipenem resistance through conjugation.

**Conclusion:**

Our findings showed that children not exposed to antibiotics can be colonized by *E. coli* isolates resistant to the commonly used antimicrobial compounds and can be a good indicator for the occurrence and prevalence of AMR in the community. These bacteria can act as a potential reservoir of AMR genes including MBL genes of pathogenic bacteria and lead to the dissemination of resistance mechanisms to other bacteria.

## Background

Since the introduction of antibiotics, antimicrobial resistance (AMR), especially amongst pathogenic bacteria has been emerging. Over the past years, AMR has been growing and becoming a global threat. Without knowing all the factors involved in AMR and the absence of plans to combat it, it is predicted that by 2050, it will be causing 10 million deaths per year [1]. The incidence of community- and hospital-acquired infections caused by multi-drug resistant (MDR) bacteria is a cause of concern. One of the main factors involved in this issue is massive and inappropriate use of antimicrobial drugs that can be lead to resistance in pathogenic bacteria [2]. On the other hand, AMR may be acquired through horizontal gene transfer between bacteria. In this case, commensal bacteria are of particular importance.

The gastrointestinal tract of mammals has a large number of bacteria as the normal commensal flora. *Escherichia coli*, a member of the *Enterobacteriaceae* family, is one of the most important bacteria of the gut microflora [3]. Commensal *E. coli* represents a reservoir of AMR; therefore, it can transfer resistance genes to pathogenic microorganisms. *E. coli* is often used in the incidence studies of antibiotic resistance in commensal bacteria [4]; also, it has been regarded as a good indicator for the surveillance and spread of AMR among pathogens [5]. In 1968, it was reported that commensal *E. coli* strains with multiple transferable antimicrobial resistance genes were isolated from feces of some infants in Dublin, Ireland [6]. The infants had not been hospitalized before and had not received antibiotics. Afterwards, the presence of resistant *E. coli* strains in children was investigated in different countries and found that the frequency of resistance varies in different regions [7].

Carbapenems including imipenem, meropenem, and ertapenem are often reserved as the last-line antibiotics against MDR Enterobacteriaceae clinical isolates, because they are stable even in the presence of extended-spectrum β-lactamases (ESBLs) and AmpC enzymes [8]. However, some bacteria have been found to be resistant to carbapenems. The mechanisms of carbapenem resistance (CR) in Enterobacteriaceae are complex and mostly attributed to the production of acquired metallo-β-lactamases (MBLs) [8]. The carbapenem-hydrolyzing enzymes fall into the three classes of A, B, and D of the Ambler classification of ESBLs. Classes A and D are serine-active carbapenemase and include *Klebsiella pneumoniae* carbapenemase (KPC) enzymes and OXA-48 group, respectively. MBLs, on the other hand, are in class B and are divided into the three main groups of IMP, VIM, and NDM enzymes [9]. Carbapenemases and particularly MBLs are the most important ESBLs because they are able to hydrolyze all beta-lactams including carbapenems, except monobactams. Most MBL-producing strains are also resistant to fluoroquinolones and aminoglycosides [10]. MBL-encoding genes are usually carried by mobile genetic elements that facilitate horizontal gene transfer (HGT) between bacteria and harbor a great ability to spread [11, 12]. MBL-producing bacteria are regarded as the most important nosocomial pathogens, and further spread of them in the healthcare settings will pose a serious global threat in the future [13]. Therefore, active surveillance is needed to detect the prevalence and incidence of MBL-producing bacteria in the community and help prevent the spread of these organisms [14].

To the best of our knowledge, there are no reports regarding the frequency of AMR in commensal *E. coli* isolates among children or at community level in Iran. Most studies have been conducted on pathogenic strains. Also, most studies performed on the prevalence of AMR in commensal strains are related to the phenotypic evaluation of antibiotic resistance, and the identification of resistance genes has been reported in a small number of studies [15]. The aim of this study was to investigate the AMR frequency at the community level and the presence of MBL-producing *E. coli* strains and MBL genes in fecal samples of healthy children aged < 3 years.

## Results

### Antimicrobial resistance and ESBL-production

A total of 412 *E. coli* strains were isolated from 430 children aged under 3 years old. Eighteen babies had no *E. coli* in their stools and all of them were under 6 months old. Out of the 412 isolates, 211 and 201 *E. coli* strains were related to Fars and Khuzestan provinces, respectively. Resistance pattern of the isolates is shown in Table 1. At least 226 (54.9%) isolates were resistant to one or more than one antibiotic. MDR profile was observed in 149 (36.2%) isolates. The most resistance was observed against ampicillin (44.2%), followed by cefotaxime (36.7%) and trimethoprim-sulfamethoxazole (32.3%). The most effective antibiotics were meropenem, imipenem, and gentamycin with the susceptibility rates of 98.5, 98.5, and 93.9% respectively. The frequency of resistance against 10 antibiotics including nalidixic acid, ampicillin, tetracycline, kanamycin, cefotaxime, ceftazidime, streptomycin, trimethoprim-sulfamethoxazole, ciprofloxacin, and gentamycin was significantly higher in Khuzestan province than in Fars province (*P* < 0.001). The overall frequency of ESBL-producing strains was 11.7% of all commensal *E. coli* isolates. The lowest resistance rates were found in *E. coli* strains isolated from children under 6 months old. The chance of the resistance to chloramphenicol, nalidixic-acid, ampicillin, tetracycline, cefotaxime, ceftazidime, streptomycin, and sulfamethoxazole-trimethoprim antibiotics and also MDR increased with age (*P* < 0.05) (Table 2). Commensal *E. coli* isolates showed a similar resistance pattern to all the tested antibiotics in both female and male children (Table 3).

**Table 1:** Frequency of AMR, MDR, and ESBL among commensal *E. coli* strains isolated from children in Khuzestan and Fars provinces

	CHL N (%)	NA^**a**^ N (%)	AMP ^**a**^ N (%)	TET^**a**^ N (%)	KAN^**a**^ N (%)	CTX^**a**^ N (%)	CAZ^**a**^ N (%)	STR^**a**^ N (%)	SXT^**a**^ N (%)	CIP^**a**^ N (%)	GEN^**a**^ N (%)	IMP^**a**^ N (%)	MEN^**a**^ N (%)	MDR^**a**^ N (%)	ESBL ^**a**^ N (%)
**Khuzestan**	23 (11.4)	88 (43.8)	130 (64.7)	85 (42.3)	18 (9)	100 (49.8)	82 (40.8)	81 (40.3)	101 (50.2)	51 (25.4)	13 (6.5)	4 (2)	4 (2)	108 (53.7)	31 (15.42)
**Fars**	15 (7.1)	36 (17.1)	52 (24.6)	37 (17.5)	7 (3.3)	51 (24.2)	33 (15.6)	27 (12.8)	32 (15.2)	11 (5.2)	4 (1.9)	2 (0.9)	2 (0.9)	41 (19.4)	17 (8.06)
**Sum**	38 (9.2)	124 (31.1)	182 (44.2)	122 (29.6)	25 (6.1)	151 (36.7)	115 (27.9)	108 (26.2)	133 (32.3)	62 (15)	17 (4.1)	6 (1.5)	6 (1.5)	149 (36.2)	48 (11.7)

^a^(Star): Statistically significant. *P* < 0.05; *CHL* Chloramphenicol, *NA* Nalidixic-acid, *AMP* Ampicillin, *TET* Tetracycline, *KAN* Kanamycin, *CTX* cefotaxime, *CAZ* ceftazidime, *STR* Streptomycin, *STX* sulfamethoxazole-trimethoprim, *CIP* ciprofloxacin, *GEN* Gentamycin, *IMP* Imipenem, *MEN* Meropenem, *MDR* Multi-drug resistance, *ESBL* Extended-spectrum beta-lactamases

**Table 2:** Frequency of AMR and MDR by age among commensal *E. coli* strains isolated from children in Khuzestan and Fars provinces

Age (Year)	Total number	CHL^**a**^ N (%)	NA^**a**^ N (%)	AMP^**a**^ N (%)	TET^**a**^ N (%)	KAN N (%)	CTX^**a**^ N (%)	CAZ^**a**^ N (%)	STR^**a**^ N (%)	SXT ^**a**^ N (%)	CIP N (%)	GEN N (%)	IMP N (%)	MEN N (%)	MDR^**a**^ N (%)
**0–1**	130	7 (5.4)	33 (25.4)	47 (36.1)	28 (21.5)	7 (5.4)	31 (23.8)	27 (20.8)	27 (20.8)	32 (24.6)	20 (15.4)	6 (4.6)	1 (0.8)	1 (0.8)	34 (26.1)
**1–2**	144	12 (8.3)	38 (26.4)	60 (41.7)	44 (30.5)	8 (5.5)	51 (35.4)	41 (28.5)	37 (25.7)	45 (31.2)	14 (9.7)	3 (2)	1 (0.7)	1 (0.7)	47 (32.6)
**2–3**	138	19 (13.8)	53 (38.4)	73 (52.9)	51 (37)	10 (7.2)	69 (50)	47 (34.05)	44 (31.9)	55 (40)	28 (20.3)	8 (5.8)	4 (2.9)	4 (2.9)	65 (47.1)
**CI**	–	0.382–0.919	0.570–0.968	0.582–0.948	0.537–0.916	–	0.431–0.725	0.546–0.940	0.567–0.986	0.550–0.927	–	–	–	–	1.230–2.056
**OR**	–	0.593	0.743	0.743	0.701	–	0.559	0.716	0.748	0.714	–	–	–	–	1.591
***P*-value**	–	0.019	0.028	0.017	0.009	0.521	0.001>	0.016	0.039	0.011	0.247	0.609	0.162	0.162	0.001>

^a^(Star): Statistically significant. *P* < 0.05; *OR* Odds Ratio, *CI* Confidence Interval, *CHL* Chloramphenicol, *NA* Nalidixic-acid, *AMP* Ampicillin, *TET* Tetracycline, *KAN* Kanamycin, *CTX* cefotaxime, *CAZ* ceftazidime, *STR* Streptomycin, *STX* sulfamethoxazole-trimethoprim, *CIP* ciprofloxacin, *GEN* Gentamycin, *IMP* Imipenem, *MEN* Meropenem, *MDR* Multi-drug resistance

**Table 3:** Frequency of AMR and MDR by sex among commensal *E. coli* strains isolated from children in Khuzestan and Fars provinces

Sex (N)	CHL N (%)	NA N (%)	AMP N (%)	TET N (%)	KAN N (%)	CTX N (%)	CAZ N (%)	STR N (%)	SXT N (%)	CIP N (%)	GEN N (%)	IMP N (%)	MEN N (%)	MDR N (%)
**Female** (216)	18 (8.3)	68 (31.5)	100 (46.3)	70 (32.4)	16 (7.4)	86 (39.8)	64 (29.6)	64 (29.6)	74 (34.3)	35 (16.2)	10 (4.6)	4 (2)	4 (2)	84 (38.9)
**Male (**196**)**	20 (10.2)	56 (28.6)	82 (41.8)	52 (26.5)	9 (4.6)	65 (33.2)	51 (26)	44 (22.4)	59 (30.1)	27 (13.8)	7 (3.6)	2 (0.9)	2 (0.9)	65 (33.2)
**Sum (**412)	38 (9.2)	124 (31.1)	182 (44.2)	122 (29.6)	25 (6.1)	151 (36.7)	115 (27.9)	108 (26.2)	133 (32.3)	62 (15)	17 (4.1)	6 (1.5)	6 (1.5)	149 (36.2)

*CHL* Chloramphenicol, *NA* Nalidixic-acid, *AMP* Ampicillin, *TET* Tetracycline, *KAN* Kanamycin, *CTX* cefotaxime, *CAZ* ceftazidime, *STR* Streptomycin, *STX* sulfamethoxazole-trimethoprim, *CIP* ciprofloxacin, *GEN* Gentamycin, *IMP* Imipenem, *MEN* Meropenem, *MDR* Multi-drug resistance

### MBL-producing bacteria

Disc diffusion and MIC tests showed that six isolates were resistant to imipenem; therefore, these isolates were investigated for MBL production. CDT and DDST were performed to investigate the MBL production of isolates. Also, mCIM, eCIM, and PBA disc tests were used to differentiate MBLs from serine carbapenemases or class A KPC carbapenemases. Out of six isolates, three were positive by CDT or DDST, while one *E. coli* isolate was negative by CDT and DDST, and it was positive by mCIM and eCIM. As a result four isolates were considered as MBL-producers.

### Carbapenemase production

Two isolates were negative for MBL production; therefore, other mechanisms that could be related to imipenem resistance were investigated. PBA disc test showed that one of these two isolates had class A KPC carbapenemase. The other isolate that was negative by the PBA disc test was positive by mCIM and negative by eCIM; therefore, this isolate had serine carbapenemase or other mechanisms for resistance to imipenem.

### Molecular identification of MBLs and carbapenemase genes

The imipenem-resistant isolates were investigated for the presence of MBL genes. The *bla*_NDM_ and *bla*_VIM-2_ genes were detected in two and one isolates, respectively. All the three isolates were also phenotypically positive for MBL production. One of the isolates did not have any of the eight MBL genes. The isolates were also investigated for the presence of the three main carbapenemase genes, including *bla*_KPC_, *oxa*-23, and *oxa*-48. These genes were not detected in any of the imipenem-resistant isolates. The characteristics of the isolates are shown in Table 2. The sequence of the *bla*_NDM-1_ gene was submitted to the GenBank nucleotide sequence database under the accession number MT260405. BLAST sequencing showed that the *bla*_NDM-1_ genes were related to NDM-5 variant.

### Plasmid profiling and conjugation

Plasmid profiling of the isolates showed the presence of 10 differently sized plasmids, from approximately 2 to 40 kb. All 6 isolates presented with different plasmid profiles. However, the bands of 12 kb and 16 kb were present in common in 3 and 2 isolates, respectively. Five isolates were able to transfer their plasmids; however, not all plasmids could be transferred by conjugation (Table 4).

**Table 4:** Pattern of antimicrobial resistance, MBLs genes, carbapenemase genes, and phenotypic characterization of commensal *E. coli* isolates resistant to imipenem

No of isolate	Age (month)	CHL	NA	AMP	TET	KAN	CTX	CAZ	STR	STX	CIP	GEN	IMP	MEN	MDR	*bla*_NDM_	*bla*_SIM_	*bla*_SPM-1_	*bla*_GIM_	*bla*_IMP-1_	*bla*_IMP-2_	*bla*_VIM-1_	*bla*_VIM-2_	*bla*_KPC_	*oxa-23*	*oxa-48*	DDST	CDT	PBA	MHT	mCIM	eCIM	MBL	Transconjugation	MIC	Size of plasmids (kb)
260	12	R	R	R	S	R	R	R	R	R	R	S	R	R	**+**	**+**	**–**	**–**	**–**	**–**	**–**	**–**	**–**	**–**	**–**	**–**	+	–	–	–	+	+	+	+	6	3P > 23
271	36	R	S	R	S	S	R	R	S	S	S	S	R	R	**+**	**–**	**–**	**–**	**–**	**–**	**–**	**–**	**+**	**–**	**–**	**–**	–	+	–	–	+	+	+	+	6	2P > 23
335	35	S	R	R	R	R	R	R	R	R	R	S	R	R	**+**	**+**	**–**	**–**	**–**	**–**	**–**	**–**	**–**	**–**	**–**	**–**	+	–	–	–	+	+	+	+	5	12, 3P > 23
175	22	S	R	S	R	R	R	S	R	S	I	I	R	R	**+**	**–**	**–**	**–**	**–**	**–**	**–**	**–**	**–**	**–**	**–**	**–**	–	–	–	–	+	–	–	–	6	16, 12, 1P > 23
176	36	S	R	R	S	S	R	R	R	S	S	S	R	R	**+**	**–**	**–**	**–**	**–**	**–**	**–**	**–**	**–**	**–**	**–**	**–**	–	–	–	–	+	+	+	+	6	16, 12
317	31	S	R	R	R	I	R	R	R	R	R	S	R	R	**+**	**–**	**–**	**–**	**–**	**–**	**–**	**–**	**–**	**–**	**–**	**–**	–	–	+	–	+	–	–	+	6	5, 14

*CHL* Chloramphenicol, *NA* Nalidixic-acid, *AMP* Ampicillin, *TET* Tetracycline, *KAN* Kanamycin, *CTX* cefotaxime, *CAZ* ceftazidime, *STR* Streptomycin, *STX* sulfamethoxazole-trimethoprim, *CIP* ciprofloxacin, *GEN* Gentamycin, *IMP* Imipenem, *MEN* Meropenem, *MDR* Multi-drug resistance, *ESBL* Extended-spectrum beta-lactamases, *DDST* Double disc synergy test, *CDT* Combined disc test, *PBA* Phenylboronic acid, *MHT* Modified Hodge test, *mCIM* Modified carbapenem inactivation method, *eCIM* EDTA-CIM, *MBL* metalo-beta-lactamase, *MIC* Minimum inhibitory concentration, *P* plasmid

## Discussion

According to the reports by world health organization (WHO), AMR is common in many countries. However, an exact estimation of the extent of the problem and the economic losses caused due to AMR is not available in Iran. WHO reports that Iran is categorized as a country with resistance to more than five antimicrobial categories per each requested bacterium and has a high prevalence of AMR in the selected pathogenic bacteria [16]; however, there is no exact information on AMR in the commensal isolates in the country. In this study, to evaluate the frequency of AMR in the community, infants and children under 3 years old were investigated. Fetuses have sterile intestines, and they receive microorganisms from their mothers at birth. After a few minutes, the gastric content of the neonate is influenced by the received flora from the mother. Gradually, infants receive intestine flora from family members, environment, water, and food and normal intestine flora forms before 3 years old [7]. Therefore, the commensal flora of this group often has not been directly exposed to antibiotics. That is why this age group was chosen and children received antibiotics were excluded from the study.

For the first time in 1966, AMR in commensal *E. coli* from healthy community members was reported [17] and further studies highlighted the increasing incidence of AMR in commensal *E. coli* from many countries [15, 18, 19]. *E. coli* is known as the main reservoir of resistance in fecal flora. In this study, the frequency of AMR in commensal *E. coli* isolates from two region of Iran was investigated and found that the incidence of AMR, MDR, and ESBL-producing isolates in Khuzestan Province was significantly higher as compared to Fars Province. Interestingly, according to the report by the National Committee for Rational Prescribing and use of Drugs (NCRUD) in Iran, in 2015 the highest and lowest mean numbers of prescribed antimicrobial agents were related to Khuzestan and Fars Provinces, respectively [20]. This level of prescribed antibiotics may be due to the high prevalence of infectious diseases in Khuzestan Province [21]. In other studies performed in Khuzestan, high frequencies of AMR, MDR, and ESBL in diarrheagenic *E. coli* (DEC) isolates [21] and ESBL-producing *E. coli* strains in urinary tract infections were reported [22]. However, the frequencies of AMR, MDR, and ESBL in our study were lower than those studies. Probably, communication between pathogenic and commensal bacteria and indirect exposure of commensal flora to antibiotics caused high AMR in the community. As reported by the European Antimicrobial Resistance Surveillance Network (EARS-Net), MDR among infectious *E. coli* isolates ranged from approximately 1% in 2002 to 4.8% in 2016 and 10.1% in 2018 [23]; however, in our study, MDR was found to be 36%, which is disconcerting. The carriage of commensal AMR, MDR and ESBL-producing isolates in healthy children under 3 years of age mostly reflects exposure to contamination in the family environment, water, and food rather than increased direct exposure to antimicrobial drugs [24].

Carbapenems are highly effective broad-spectrum beta-lactam antibiotics commonly used as the last-line antibiotics for the treatment of severe or high-risk antibiotic resistant Gram-negative bacterial infections. The emergence and dissemination of carbapenem-resistance mechanisms represent a global public health concern because no solutions have been found for this problem yet [25]. According to a report by Castanheira et al. the rates of CR in pathogenic Enterobacteriaceae increased from 0.6% in 1997–2000 to 2.9% in 2013–2016. In our study, the rate of CR in commensal *E. coli* strains was 1.6%; however, we do not have the exact rate of CR in pathogenic Enterobacteriacea [23].

The emergence of MBL-producing isolates is alarming since they carry mobile genetic elements with great ability to spread; therefore, early detection of these isolates, particularly their reservoir, is crucial to prevent their inter- and intra-care setting dissemination and establish suitable antimicrobial therapies [12]. To find MBL-producing isolates, we used different tests including CDT, DDST, MHT, mCIM, and eCIM because no perfect test has been introduced to identify all types of MBLs. Using mCIM in combination with eCIM, we could detect four MBL-producing isolates. According to the CLSI-2018, MHT is no longer considered a reliable phenotypic method for carbapenemase detection and other methods such as the CarbaNP and mCIM have taken its place because MHT cannot detect some carbapenemase-producing isolates including NDM-producing strains [12]. In the present study, *bla*_NDM_-positive isolates were not detected by MHT. Castanheira et al. collected pathogenic *Enterobacteriaceae* isolates from 42 countries over 20 years and found that the rates of MBL-carrying isolates increased from 4.3% of the CR isolates in 2007 to 2009 to 12.7% from 2014 to 2016 [23], while in the present study 66% of commensal CR isolates were detected as MBL-producing. PCR results showed that two and one isolates had the ability to produce NDM and VIM-2, respectively; however, one MBL-producing isolate was negative for all the investigated genes. NDM has worldwide distribution and is the most common MBL in the Enterobacteriaceae family [25]. Based on the report by Castanheira et al.*,* the dissemination of isolates carrying *bla*_NDM_ caused increased CR among Enterobacteriaceae isolates from 2014 to 2016 [26]. For the first time in Iran, NDM was detected in *K. pneumonia* in 2013 [27]. Eyvazi et al. reported NDM-producing *E. coli* isolates in 2017 [28] and NDM-producing *Pseudomonas aeruginosa* was isolated from filters of household water treatment systems in Ahvaz, Khuzestan, Iran [29]. Probably in Khuzestan, some of the resistance mechanisms are transported through the water from resistant environmental bacteria to the intestinal commensal flora*.* The spread of isolates carrying *bla*_VIM_ in Italy and Greece led to increased CR among the European countries in 2005 [23]. There are some reports in Iran on the detection of *bla*_VIM-2_ in clinical isolates such as hypervirulent *Klebsiella pneumonia* or pathogenic *P. aeruginosa* [30, 31]*.* However, we did not find any reports regarding the detection of MBL genes among commensal *E. coli* isolates. The present results showed that the commensal *E. coli* isolates can harbor MBL resistance plasmids and transfer them through conjugation. All of the transconjugative colonies were resistant to imipenem; however, not all of the plasmids were transferred. The *bla*_NDM_ genes are often carried by plasmids; therefore, they can easily move to other bacteria through HGT, which increases the probability of the emergence of antimicrobial resistant strains of pathogenic bacteria [32] as seen in the present study. The bacteria that synthesize NDM-1 are highly resistant to all antibiotics, including carbapenems and aminoglycosides [32]; while, two NDM-producing isolates in our research were sensitive to gentamicin an aminoglycoside. NDM-1 has several variants and the one identified in our research was related to an NDM-5 variant. This variant has a greater hydrolytic activity than NDM-1 toward carbapenems, and cephalosporins including cefotaxime, cephalotin and ceftazidime [33].

Out of six CR isolates, one was found phenotypically positive by PBA and mCIM tests and considered class A KPC carbapenemase. However, we could not identify the carbapenemase gene of this isolate. One CR isolate was positive only by mCIM. The resistance of this isolate is probably as class A or D of ambler classification, although this isolate was negative by the general primers of *bla*_KPC_, *oxa48*, and *oxa23*. Resistance to carbapenems can also be due to non-carbapenemase-mediated mechanisms, such as hyper production of a β-lactamase, typical AmpC β-lactamase, combination of ESBLs or AmpCs with porin mutations, and reduced membrane permeability [11, 25, 34].

Despite the development of new tests to identify carbapenemase and MBL-producing isolates, complete tests to identify all types of these resistances have not yet been introduced; therefore, we were unable to detect the exact mechanism of imipenem resistance in one isolate. The other limitation of this study was that ESBL genes were not investigated in resistant isolates. Resistance against even one antibiotic in the commensal flora is worrying as it highlights the dissemination of AMR and acts as a potential reservoir of resistance genes of pathogens [2]. The resistance against carbapenems, the last-line antibiotic against MDR Gram-negative bacteria, is increasing worldwide and dramatic changes in the epidemiology of carbapenemases, especially MBLs, are observed [23].

## Conclusion

This study showed healthy children under 3 years old carry bacteria resistant to antibiotics even in the absence of direct antimicrobial selection. The occurrence of AMR can vary in different geographical regions; therefore, knowing regional AMR, especially in the commensal flora, provides important information for empiric antimicrobial therapy decision-making. The emergence of the mutant strain NDM producing commensal *E. coli* isolates that are not associated with any infectious diseases has thrown light on the fact that these isolates can act as a reservoir of antibiotic resistance genes and transfer them among commensal microorganisms, including into pathogens. If antimicrobial resistance genes are transferred and spread to pathogenic strains, it will be very difficult to control and prevent diseases caused by these pathogens. Therefore, it is necessary to monitor the prevalence of AMR in both commensal and pathogenic bacteria.

## Methods

### Sampling and cultivation

Based on the 95% confidence interval (CI), margin error of 5%, and prevalence of 0.65 [21], the sample size was calculated as 373. This sample size was adjusted to 410 after considering a 10% non-response rate. We decided to be conservative and collected a total of 430 samples from the provinces of Khuzestan and Fars from October 2017 to December 2018. Khuzestan Province is located in southwest of Iran, and Fars is located in south of Iran. Simple random sampling was used for the selection of subjects. Children who were referred to health centers for vaccination were randomly selected using Excel, and their stool samples were collected by sterile swap. Swaps were placed in a transport medium and were immediately transported to the laboratory on ice. The studied population included healthy infants and children under 3 years old who did not show any symptoms of diseases, especially gastrointestinal diseases such as vomiting, diarrhea, nausea, stomach ache, and abdominal cramps; also, they had not received antibiotics at least during the past 4 months. Unhealthy children or those who had received antibiotics were excluded from the study. Samples whose bacterial culture was negative, were excluded from statistical analysis. The study protocol conformed to the ethical guidelines of the Declaration of Helsinki (No: EE/99.24.3.88342/scu.ac.ir). Because the participants were children under 3 years old, parents or guardian were asked to read, accept and sign an informed consent form before any information was collected. Then cultured on MacConkey (MC) agar (Merck; Frankfurt, Germany) and Eosin Methylene Blue (EMB) agar (Merck; Frankfurt, Germany) for the isolation of *E. coli* strains. People usually carry a predominant *E. coli* strain that forms more than half of the isolated colonies from their fecal samples [35, 36] and therefore, from each cultured sample, one isolate was randomly selected for further analysis. The isolates were confirmed by standard biochemical tests and the highly specific *E. coli* universal stress protein A (*uspA* gene) was detected using PCR as described by Chen and Griffiths [37].

### Antimicrobial resistance and ESBL production

The pattern of antibiotic susceptibility of the isolates was determined by the standard Kirby-Bauer disc diffusion method according to Clinical and Laboratory Standards Institute (CLSI-2016) [38]. The antibiotic discs included chloramphenicol (30 μg), nalidixic acid (30 μg), ampicillin (10 μg), tetracycline (30 μg), kanamycin (30 μg), cefotaxime (30 μg), ceftazidime (30 μg), streptomycin (10 μg), trimethoprim-sulphmetoxazole (23.75–1.25 μg), ciprofloxacin (5 μg), gentamicin (10 μg), meropenem (10 μg) and imipenem (10 μg). The minimum inhibitory concentration (MIC) of imipenem-resistant isolates was determined as described by CLSI-2016 [38]. According to the CLSI guidelines, MIC breakpoint for imipenem resistance *E. coli* isolates was defined ≥4 μg/ml. MDR was defined as resistance to three or more different antibiotic families. To evaluate ESBL production in the isolates, synergy double disc (SDD) method was performed using ceftazidime (30 μg) and cefotaxime (30 μg), alone and in combination with clavulanic acid (10 μg). A ≥ 5-mm increase in zone diameter for either cefotaxime or ceftazidime in combination with clavulanic acid versus the zone diameter of the agents when tested alone was considered as ESBL production [38].

### Combined disc test (CDT) and the double disc synergy test (DDST)

The two methods of CDT and DDST were adopted to detect MBL-producing isolates. For the CDT, Mueller-Hinton agar (MHA) plate was inoculated by 0.5-McFarland test isolate. Then, two discs of imipenem and imipenem containing 10 μL of 0.5 M ethylenediaminetetraacetic acid (EDTA) were placed on the occulted plate. The plates were incubated at 35 °C for 16–18 h. An increase in zone inhibition of equal or more than 7 mm around the imipenem-EDTA disc compared to imipenem alone was considered MBL-positive [12].

To perform DDST, the two discs of imipenem and blank disc with a distance of 15 mm were placed on MHA plate that was inoculated by 0.5-McFarland standard. Then, blank disc was impregnated with 10 μL of 0.5 M EDTA. The plates were incubated at 35 °C for 16–18 h. The enhancement of the inhibition zone or appearance of a phantom zone between the imipenem and EDTA discs was considered positive for MBL production [12].

### Modified carbapenem inactivation method (mCIM) and EDTA-CIM (eCIM)

mCIM was performed to detect carbapenemases in *E. coli* isolates and then eCIM was used together with mCIM to differentiate MBLs from serine carbapenemases. Briefly, 1 μL loopful of bacteria was emulsified in 2 mL of TSB; then, one meropenem (10 μg) disc was added to the emulsion and incubated at 35 °C for 4 h. An MHA plate was inoculated with a 0.5-McFarland suspension of meropenem-susceptible *E. coli* ATCC25922. Meropenem disc was removed from TSB-meropenem disc suspension and placed on the MHA plate inoculated with the *E. coli* ATCC 25922 indicator strain. Following the incubation of MHA plate at 37 °C for 18–24 h, the zone of inhibition was measured. The isolates that showed a zone inhibition below 15 mm or the presence of pinpoint colonies within a 16–18 mm zone were considered as carbapenemase-positive. For eCIM, all the steps were similar to mCIM, except that EDTA was added to TSB to obtain a final concentration of 5 mM EDTA. The isolates that produce an increase of ≥5 mm in zone diameter for eCIM compared to mCIM were considered MBL-positive [26].

### Modified Hodge test (MHT)

The MHT test was performed for the phenotypic detection of carbapenemase production. Briefly, a 0.5-McFarland suspension of *E. coli* ATCC25922 was prepared and diluted 1:10 in saline. An MHA plate was inoculated with the indicator *E. coli*; then one meropenem (10 μg) disc was placed at the center of the plate. Subsequently, test organisms were cultured on the plate in a straight line from the edge of the disc in a length of 25 mm. The enhanced growth of the indicator *E. coli* strain towards the carbapenem disc was considered as a positive result for carbapenemase production [34].

### Phenylboronic acid (PBA) disc test

PBA disc test was performed for the phenotypic differentiation of MBLs and class A KPC carbapenemases. PBA was dissolved in dimethyl sulfoxide (DMSO) to obtain a final concentration of 20 mg/ml. Then, 20 μl (400 μg PBA) of the solution was dispensed onto the meropenem disc. The standard disc diffusion method was performed for the isolates by meropenem (10 μg) disc with and without PBA. After incubation of the plates at 37 °C for 18 h, the diameter of the inhibition zones was measured. An increase of ≥5-mm in zone diameter for PBA-meropenem compared to plain meropenem was considered KPC carbapenemase [8, 39].

### PCR amplification

Total DNA was isolated using the boiling method. MBL genes including *bla*_IMP-1_, *bla*_IMP-2_, *bla*_VIM-1_, *bla*_VIM-2_, *bla*_SPM-1_, *bla*_NDM-1_, *bla*_SIM_, and *bla*_GIM_ were amplified. PCR conditions were followed as described previously [13, 40–43]. Additionally, strains were tested for *bla*_KPC_, *oxa*-23, and *oxa*-48, the three main genes of carbapenemases as described in the corresponding references (Table 5) [44–46]. The PCR products were subjected to Sanger sequencing (BIONEER Company; Daejeon; Korea). Their nucleotide sequences were analyzed with software available from the National Centre for Biotechnology Information (www.ncbi.nlm.nih.gov).

**Table 5:** Names and sequences of primers used in this study

Primer name	Sequence (5′ → 3′)	References
*bla*_IMP-1_F	ACCGCAGCAGAGTCTTTGCC	[13]
*bla*_IMP-1_R	ACAACCAGTTTTGCCTTACC	
*bla*_IMP-2_F	GTTTTATGTGTATGCTTCC	[13]
*bla*_IMP-2_R	AGCCTGTTCCCATGTAC	
*bla*_VIM-1_F	AGTGGTGAGTATCCCGACAG	[39]
*bla*_VIM-1_R	ATGAAAGTGCGTGGAGAC	
*bla*_VIM-2_F	ATGTTCAAACTTTTGAGTAAG	[41]
*bla*_VIM-2_R	CTACTCAACGACTGAGCG	
*bla*_SPM-1_F	GCGTTTTGTTTGTTGCTC	[13]
*bla*_SPM-1_R	TTGGGGATGTGAGACTAC	
*bla*_GIM_F	TCGACACACCTTGGTCTG	[40]
*bla*_GIM_R	AACTTCCAACTTTGCCAT	
*bla*_SIM_F	TACAAGGGATTCGGCATCC	[40]
*bla*_SIM_R	TAATGGCCTGTTCCCATG	
*bla*_NDM_F	GGCGGAATGGCTCATCACGA	[43]
*bla*_NDM_R	CGCAACACAGCCTGACTTTC	
*Bla*_KPC_-F	‘ATGTCACTGTATCGCCGTCT	[44]
*bla*_KPC_-R	‘TTTTCAGAGCCTTACTGCCC	
*oxa*-*23*-F	AAGCATGATGAGCGCAAAG	[45]
*oxa*-*23*-R	AAAAGGCCCATTTATCTCAAA	
*oxa*-*48*-F	TTGGTGGCATCGATTATCGG	[46]
*oxa*-*48*-R	GAGCACTTCTTTTGTGATGGC	
*uspA*-F	CCGATACGCTGCCAATCAGT	[37]
*uspA*-R	ACGCAGACCGTAGGCCAGAT	

### Plasmid profiling

The resistant imipenem isolates were investigated as to their plasmid content. Plasmid DNA was extracted by an alkaline lysis method [47]. The products were electrophoresed on 1% agarose gels. The size of the plasmid bands were determined using a molecular weight marker, made from a lambda/*Hind* III digest.

### Conjugation experiment

The resistant imipenem isolates were conjugated with an imipenem sensitive, lactose-negative, enteroinvasive *E. coli* (EIEC) plasmid-free strain. The overnight cultures of the donors and recipient *E. coli* isolates were mixed in a ratio of 1:10 in nutrient broth and incubated for 48 h at 37 °C. Then, the mixtures were spread on MacConkey agar containing imipenem (4 μg/mL) and incubated overnight at 37 °C. The lactose-negative, imipenem resistant isolates were analyzed for the presence of plasmids [47].

### Statistical analysis

SPSS software (v.22.0) was used for data analysis. A χ2 test or Fisher’s exact test was used to determine the statistical significance of the data. A *P* value of < 0.05 was considered as statistically significant. The relations between the AMR profiles and the age of children were described using logistic regression model with 95% CI.

## Data Availability

The datasets used and/or analyzed during the current study are available from the corresponding author on reasonable request. Sequence data of this project have been deposited in the GenBank of the National Center for Biotechnology Information (NCBI) under the accession number MT260405.

## Abbreviations

AMR: Antimicrobial resistance
MDR: Multi-drug resistant
ESBLs: Extended-spectrum β-lactamases
CR: Carbapenem resistance
MBLs: Metallo-β-lactamases
KPC: *Klebsiella pneumoniae* carbapenemase
HGT: Horizontal gene transfer
MC: MacConkey
EMB: Eosin Methylene Blue
CLSI: Clinical and Laboratory Standards Institute
SDD: Synergy double disc
CDT: Combined disc test
DDST: Double disc synergy test
mCIM: Modified carbapenem inactivation method
eCIM: EDTA-CIM
MHT: Modified Hodge test
PBA: Phenylboronic acid
DMSO: Dimethyl sulfoxide
EIEC: Enteroinvasive *E. coli*
WHO: World health organization
DEC: Diarrheagenic *E. coli*
